# Efficacy of Entrepreneurs' Psychological Capital on the Performance of New Ventures in the Development of Regional Economy in the Greater Bay Area

**DOI:** 10.3389/fpsyg.2021.705095

**Published:** 2021-09-28

**Authors:** Hongbo Chen, Yitao Tao

**Affiliations:** China Center for Special Economic Zone Research, Shenzhen University, Shenzhen, China

**Keywords:** Greater Bay Area, psychological capital, efficacy, new ventures, performance

## Abstract

The study aims to analyze the relationship between the psychological capital and enterprise performance of the entrepreneurs and provide direction for the optimization of regional economic development and talent introduction mechanism. First, the theoretical basis is established from regional economic development, innovative talent introduction, and individual psychological capital theory, according to which the questionnaire survey is designed, and four dimensions of self-efficacy (C), wish (W), toughness (T), and optimism (O) are selected to set the questions on psychological capital and innovation performance. Then, following statistical analysis, the characteristics of the new ventures are revealed. Finally, based on the empirical research model, the hypotheses are put forward based on psychological capital and its four subdimensions, and the relationship between the efficacy of the psychological capital and the performance of new ventures of the entrepreneurs is revealed through regression analysis. The results show that the proportion of the computer and education industry in the new ventures reaches 67.7%, and the proportion of enterprises with 1–5 entrepreneurial years reaches 78%. Meanwhile, the proportion of first-time entrepreneurship is 60.5%, and the proportion of entrepreneurs motivated by their ideal reaches 35.8%. The new entrepreneurs have a good grasp of the market situation, and the overall operation condition is stable. Most of them originate from micro and small enterprises (MSE). Psychological capital has a great influence on individual entrepreneurship. The empirical results show that the psychological capital of entrepreneurs has a positive effect on the performance of new ventures. Except for the subdimension of O, the other three subdimensions (C, T, and W) of psychological capital have a positive effect on the performance of new ventures, and psychological capital as a whole has the greatest impact. The results provide a reference for the relationship between the performance improvement of new ventures and regional economic development.

## Introduction

According to the Belt and Road initiative, deepening cooperation with Hong Kong, Macao, and Taiwan has become an important part of the construction of the Greater Bay Area (Guangdong–Hong Kong–Macao Greater Bay Area) (Lu et al., 2018; Guo et al., 2021). Due to its special geographical location, the region has a great influence on the surrounding economy. Meanwhile, given the factors, such as diverse regional cultures, different laws and regulations, and distinct talent policies, the overall regional management has been complicated. Industrial agglomeration has attracted more talents, but the outflow of talents is also very prominent (Liu et al., 2017; Lopez-Navarro et al., 2018; Taseiko et al., 2019). Innovation drives economic development, and talent is the core of innovation, especially for new ventures (Bajwa et al., 2017; Michela, 2017). In the new ventures, entrepreneurs have a great impact on enterprise development and improvement. At present, many scholars have studied the relationship between entrepreneur capital and performance. To analyze the influence of psychological capital on deviant innovation behaviors, the correlation between job remodeling and deviant innovation, psychological capital, and work values are statistically analyzed, and the relationship between psychological capital and deviant innovation behaviors are studied based on the analytic hierarchy process. The results show that there is a significant correlation between entrepreneurial performance and enterprise performance (Xu and Zhao, 2020). Here, multiple regression is chosen to analyze the correlation between the innovation behavior of enterprise employees and the alternation and the psychological capital of enterprise leaders based on a questionnaire survey (QS). The results indicate that self-efficacy (C), contribution, and toughness (T) of employees are positively correlated with innovation behaviors, and wish (W), emotion, loyalty, and optimism (O) are positively correlated with innovation behavior of the employee, whereas emotion, contribution, and loyalty play a mediating role in the psychological capital of enterprise leaders and their innovation behaviors (Li et al., 2020). The relationship among children and the psychological structure, mental health symptoms, and subjective well-being of adolescents is analyzed through indices, such as W, C, O, anxiety, and depression, implying that there is a significant correlation between the psychological structure of the respondents and their psychological symptoms. Positive psychology can alleviate poor mental health and promote happiness (Finch et al., 2020).

Here, the impact of the psychological capital of entrepreneurs is studied on the performance of new ventures of regional economic development in the Greater Bay Area. To explore whether the psychological capital of entrepreneurs has an impact on the performance of new ventures, data are collected through QS, and the relationship between the two is revealed through regression analysis. The innovation is to design the QS through a combination of regional economic development, innovative talent introduction, and individual psychological capital, making the survey data fully supportive of the research results. It is hoped that the results can provide some reference for the development of new ventures and the improvement of enterprise performance.

## Theories and Research Methods of Psychological Capital

### Development and Innovative Talent Introduction in the Greater Bay Area

Under the reform and opening up, the construction of the Greater Bay Area has become one of the key development strategies and has developed into a large-scale industrial complex (Cao et al., 2019; Hasan et al., 2020; Wang et al., 2021a). In recent years, employment in the Greater Bay Area is on the rise. The rapid development of private enterprises has provided many jobs in the region, and the overall employment structure has improved significantly. The Greater Bay Area shows a strong attraction in terms of industrialization and resource allocation. The characteristics and experience of the regional economic development can play a positive role in accelerating industrial reformation and innovation. Professional skilled talents have been brought into the Greater Bay Area, increasing human resources and intensifying talent competitiveness. The development of the regional economy depends on talents. Talent is the foundation and core of innovation and entrepreneurship (Ghisellini et al., 2018; Diner et al., 2020).

In terms of talent introduction and innovation, the Greater Bay Area has introduced a series of policies, such as housing provision and subsidies for master and doctoral talents, to provide support for innovation and entrepreneurship. Small and medium-sized enterprises have played an important role in the development of the Greater Bay Area. Meanwhile, the problem of talent outflow is still very serious, bringing great losses to the performance of enterprises (Keeeun et al., 2017; Andersson et al., 2018). In general, talent introduction has a long way to go in the Greater Bay Area, and many problems have surfaced, such as imperfect structure, low international talent aggregation, insufficient platform optimization, and imperfect introduction mechanism. Thus, enterprises should clarify the demand for high-level talents and diversify talent introduction mechanisms. The development of the regional economy depends greatly on the cultivation of talents, and the influence of entrepreneurs on new ventures cannot be overemphasized. The performance of enterprises will be comprehensively impacted by social capital, human capital, and psychological capital. Development is the first priority, talent is the primary resource, and innovation is the core driving force. At present, international competition is fierce, and national competitiveness reflects the technological strength of countries, which is ultimately determined by innovative talents (Rojas-Torres and Kshetri, 2019). Human is the most critical factor in scientific and technological innovation, and innovative undertakings call for innovative talents. To lead the world in scientific and technological innovation, China must excavate talents in innovative practices, cultivate talents in innovative activities, and aggregate talents in innovative undertakings. Obviously, talents play a vital role in national development, and it is essential to introduce innovative talents, and talent work should focus on promoting the transformation from demographic dividend to talent dividend. However, China is facing problems, such as shortage of talents and difficulty in the introduction of new talents. The reservation and utilization of talents are still insufficient. At present, many local government-mandated policies have been promulgated to attract more talents, including settlement and housing subsidies. Thus, it is never too late to start. Here, the psychological capital of entrepreneurs is mainly studied.

### The Theory of Entrepreneurs' Psychological Capital

The psychological capital has been derived from positive psychology. Different scholars have different understandings of psychological capital. Currently, there are three popular psychological capital theories. Specifically, they are trait theory, state theory, and comprehensive theory (Stratman and Youssef-Morgan, 2019; Wang et al., 2021b). In the context of regional economic development in the Greater Bay Area, psychological capital is interpreted as a kind of psychological resource that can improve the performance of entrepreneurs and is a manifestation of a positive psychological state. Factors such as C, T, and O are all expressions of psychological capital (Kathrin et al., 2018; Sui et al., 2019; Woo and Kim, 2020). The scales used for the measurement of psychological capital are diverse in elements and focuses (Han et al., 2019). The two-dimensional theory is the earliest psychological capital measurement scale proposed. The elements in the three-dimensional theory include several versions, such as a version with C, T, and O, and another version with W, T, and O, with overlapping elements. Currently, the four-dimensional measurement scale is the most widely used in psychological capital, and the version including W, C, O, and T is agreed on by most scholars. Moreover, there are other versions of the four-dimensional measurement scale for psychological capital with different elements (Alfonso et al., 2018). The psychological capital of entrepreneurs is the internal force driving the entrepreneurial process. Without W (in psychological capital), entrepreneurs will not have the passion to venture. Without C (in psychological capital), entrepreneurs will not offer wages to people in exchange for corporate residual rights to control. Lacking T (in psychological capital), entrepreneurs can neither continue to face the difficulties in the entrepreneurial process nor can they recover from setbacks or failures. Without O (in psychological capital), entrepreneurs will attribute the disadvantages of the entrepreneurial process as inherent and inevitable factors, and they cannot create an optimistic atmosphere in entrepreneurial teams and new ventures. The psychological capital of entrepreneurs runs through the entrepreneurial process and has a greater impact on entrepreneurial success than other capitals. Gao et al. (2020) studied the relationship between the psychological capital and new venture performance of entrepreneurs. The study found that compared with economic capital, human capital, and social capital, the psychological capital of entrepreneurs explains more of the performance changes of new ventures. The more uncertain the environment is, the stronger the correlation between the psychological capital and new venture performance of the entrepreneurs is. The economic capital and human capital of entrepreneurs are not found to be significant predictors of new venture performance. The social capital of entrepreneurs is a negative predictor of new venture performance, and only the psychological capital of entrepreneurs is a positive predictor of new venture performance.

### Efficacy of Psychological Capital and QS Design

The efficacy of psychological capital is realized through three different types of variables, and these three variables are the embodiment of the role of psychological capital at different levels. The first is an independent variable, which is a determining factor to the outcome variables. The second is an intermediary variable, which has an intermediary role. The third is a regulatory variable, which has a moderating effect of two different variables (Wang et al., 2018). Most research on the efficacy of psychological capital focuses on two areas, positive organizational behaviors and human resource management, where psychological capital is an independent variable. Today, the research of the efficacy of psychological capital is shifting toward entrepreneurial management. Here, the efficacy of psychological capital is analyzed in the performance of new ventures.

Subsequently, the QS method is chosen to analyze the influence of psychological capital efficacy on the performance of new ventures (Karadere et al., 2019). The QS includes the psychological capital questions and enterprise performance questions. Based on item design, the four-dimensional measurement scale is used here, and elements, such as C, T, W, and O are selected. Specifically, C can reveal the beliefs of an individual in self-cognition or behaviors, T is the embodiment of the willpower of an individual and reflects the ability to overcome difficulties, W reveals the positive state of an individual, and O reveals a positive attribution and reflects a positive mood. Accordingly, the QS is designed with five questions for each element, as shown in Table 1.

**Table 1:** Distribution and setting of psychological capital QS.

**Elements**	**Items and content**
Self-efficacy	SE1: Do you think you have the foresight and can solve a problem independently?
	SE 2: Do you think you can elaborate your work clearly in the management meeting?
	SE3: Do you think you can accomplish work objectives and thereby contribute to the strategic development of enterprises?
	SE 4: Do you think you have enough diplomatic skills?
	SE 5: Do you think you can convey effective messages to your team?
Toughness	T1: Do you think you can quickly adjust yourself in face of difficulties and continue to work enthusiastically?
	T2: Do you think you are brave enough to face any hardship and setback encountered in the work?
	T3: Do you think you can independently and liberally handle unfamiliar or new businesses?
	T4: Do you think you have been tempered by previous practice with a fearless spirit for hardship and can bravely meet difficulties?
	T5: Do you think you can cope with stress at work and handle multiple tasks concurrently?
Wish	W1: Do you think you can always find solutions to the challenges encountered at work?
	W2: Do you think you can accomplish work objectives in the best state?
	W3: Do you think you can always think of alternative solutions for work objectives?
	W4: Do you think you have made some achievements in your work?
	W5: Do you think you are getting closer to your work objectives?
Optimism	O1: Do you have the best expectations for unidentified events?
	O2: Do you think adversities are transient, and you can find solutions for adversities?
	O3: Do you think you can always look at the bright side of the work?
	O4: Do you think you are optimistic about the future of your work?
	O5: Do you think you are initiative in your current work?

The questions of entrepreneurial performance are set from two dimensions of Survival Performance (SP) and Growth Performance (GP), as shown in Table 2.

**Table 2:** Distribution and setting of enterprise performance QS.

**Dimension elements**	**Items and content**
SP	SP1: Has the enterprise been continuously operating for more than five years?
	SP2: Can the enterprise cope with the existing crises?
GP	GP1: What is the annual growth rate of sales and market share?
	GP2: What is the annual growth rate of profit?
	GP3: What is the annual growth rate of staff members?
	GP4: What is the annual growth rate of net assets?

Before the QS is issued, a presurvey is carried out to analyze the reliability and validity of the QS. The reliability is tested through Cronbach's alpha coefficient, and the validity is tested through the KMO (Kaiser–Meyer–Olkin) test and Bartlett spherical test. The QS is only used for scientific research, and it ensures that personal privacy will not be disclosed and conducted anonymously. In the presurvey, the enterprises included in an entrepreneurial incubator platform are selected. Here, employees of enterprises from different industries and different ownerships in the Greater Bay Area are recruited and investigated through the QS. The research scope focuses on multinational corporations and their subsidiaries. The distribution is reasonable, the structure is clear, and the pertinence is strong. QSs are distributed by mail, e-mail, and face-to-face. In total, 250 QSs are distributed, 245 are recovered, including 224 valid QSs, with a recovery rate of 98% and a recovery efficiency of 89.6 %, and so the recovery was good. Thus, the influence of the psychological capital efficacy of entrepreneurs on new venture performance is preliminarily analyzed under the background of regional economic development in the Greater Bay Area.

### Research Hypothesis and Data Analysis

Different from society and human capital, the psychological capital of an entrepreneur has some particularity. As a component of an individual, the psychological capital of the entrepreneur impacts enterprise performance from three aspects. First, the efficiency of entrepreneurial activities can be improved through higher individual performance. Second, enterprise performance can be improved through the job performance of employees that can be perfected through the efficacy of the psychological capital of the employees. Third, the psychological capital of entrepreneurs is positively correlated with enterprise performance. Consequently, the following hypotheses are put forward.

H1: Psychological capital of entrepreneurs has a positive effect on the performance of new ventures.
H2: Entrepreneurial C level has a positive effect on the performance of the new venture.
H3: Entrepreneurial T level has a positive effect on the performance of the new venture.
H4: Entrepreneurial W level has a positive effect on the performance of the new venture.
H5: Entrepreneurial O level has a positive effect on the performance of the new venture.

**Figure 1:**
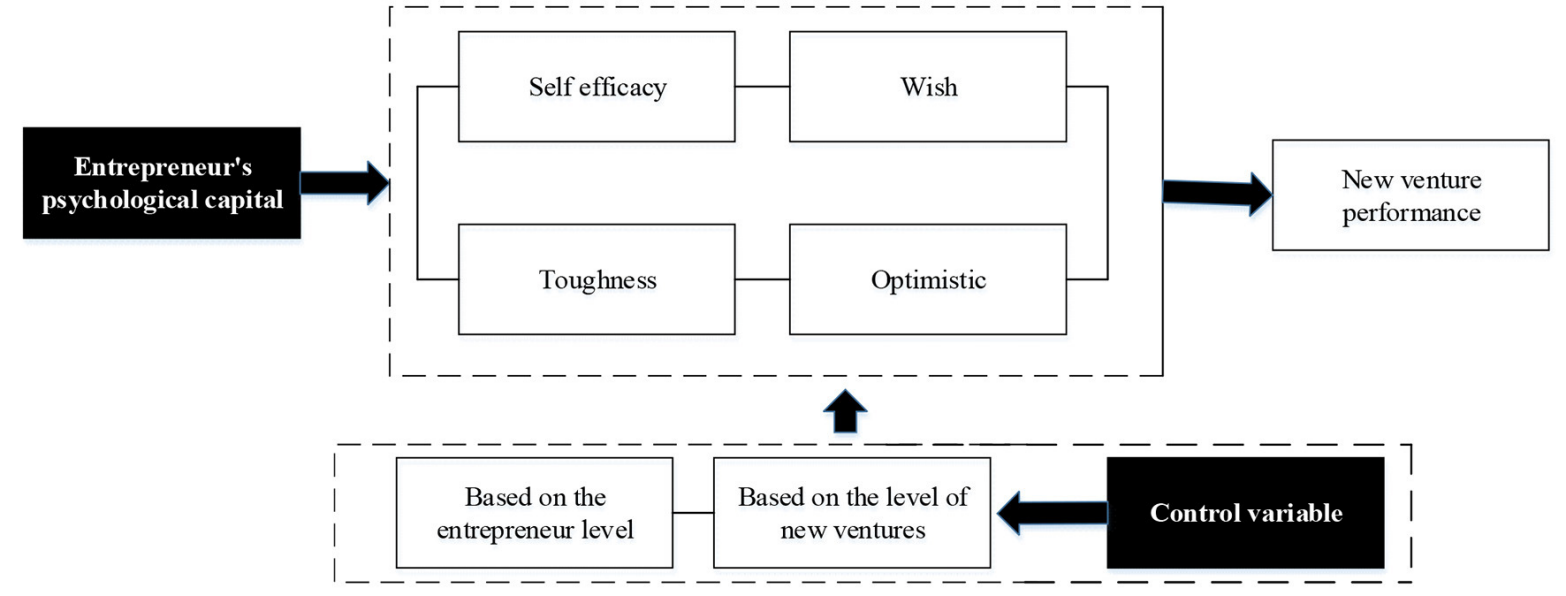
Empirical research model based on the psychological capital and new venture performance of an entrepreneur.

The empirical research model is shown in Figure 1.

Then, data analysis of the QS is completed by SPSS 25.0, including descriptive statistical analysis, regression analysis, and cluster analysis of the samples. The efficacy of psychological capital on enterprise performance is analyzed through the statistical results. In empirical research, the performance of new ventures is regarded as the result variable.

## Results of Data Analysis and Hypothesis Testing

### QS Statistical Analysis

The results of the reliability and validity test for the designed QS are shown in Table 3.

**Table 3:** Reliability and validity test results of the designed psychological capital QS.

**Reliability test**	
**Dimensions**	**Cronbach's Alpha coefficient**
Self-efficacy	0.862
Toughness	0.836
Wish	0.868
Optimism	0.848
Psychological capital	0.945
	**Validity test**
KMO	0.956
Bartlett spherical test	4633.06
Sig.	0.000

Table 3 illustrates that Cronbach's Alpha coefficients of psychological capital QS are above 0.8, proving that the reliability of the QS is good. The validity test results display that the value of KMO is above 0.8, which is suitable for factor analysis. Overall, the designed QS has good reliability and validity.

The descriptive statistical results of the QS of new venture performance are shown in Table 4.

**Table 4:** Sample statistical results of QS for new venture performance.

**Entrepreneurship-related projects**	**Specific components**	**Proportional distribution (%)**
Enterprise industry	Manufacturing	3.9
	Finance and computer software	35.4
	Service industry	20.8
	Education	32.3
	Other	7.6
Business duration	Less than a year	14.8
	1-3 years	42.5
	3-5 years	35.5
	Over 5 years	7.2
Total number of employees	Less than 10 personnel	26.5
	10-50 personnel	42.5
	50-100 personnel	28.8
	1 Over 100 personnel	2.2
Entrepreneurial experience	Once	60.5
	Twice	23.8
	More than twice	15.7
Entrepreneurial motivation	Improve living quality	26.8
	Conform to social development and improve social status	28.5
	Realize the ideal	35.8
	Other	8.9
Other project elements	Social support	16.2
	Self-efficacy, Optimism, and Toughness	44.5
	School and family	21.2
	Team collaboration	10.6
	Other	7.5

Table 4 demonstrates that the designed QS involves all walks of life. From the industrial perspective, most enterprises are from the financial industry, computer software industry, and education industry, with a cumulative proportion of 67.7%. Meanwhile, new ventures have a good grasp of the market situation and demand. From the perspective of business duration, 1- to 5-year-old enterprises account for the majority, with a cumulative proportion of 78%, suggesting that the overall operation of new ventures is stable. From the perspective of enterprise personnel composition, enterprises with 10–50 personnel account for the majority. Thus, most new ventures start from micro and small enterprises (MSE) with a small number of staff members. In terms of entrepreneurial experience and motivation, the entrepreneurs with first-time entrepreneurship account for the majority, 60.5%. Entrepreneurial motivation varies from person to person, while most entrepreneurs are motivated by their ideal, accounting for 35.8%. Among all the factors affecting entrepreneurship, the psychological capital level is the most demanded, and so it is necessary to analyze the behaviors of new ventures from the perspective of psychological capital.

### The Test on the Efficacy of Entrepreneurs' Psychological Capital

**Figure 2:**
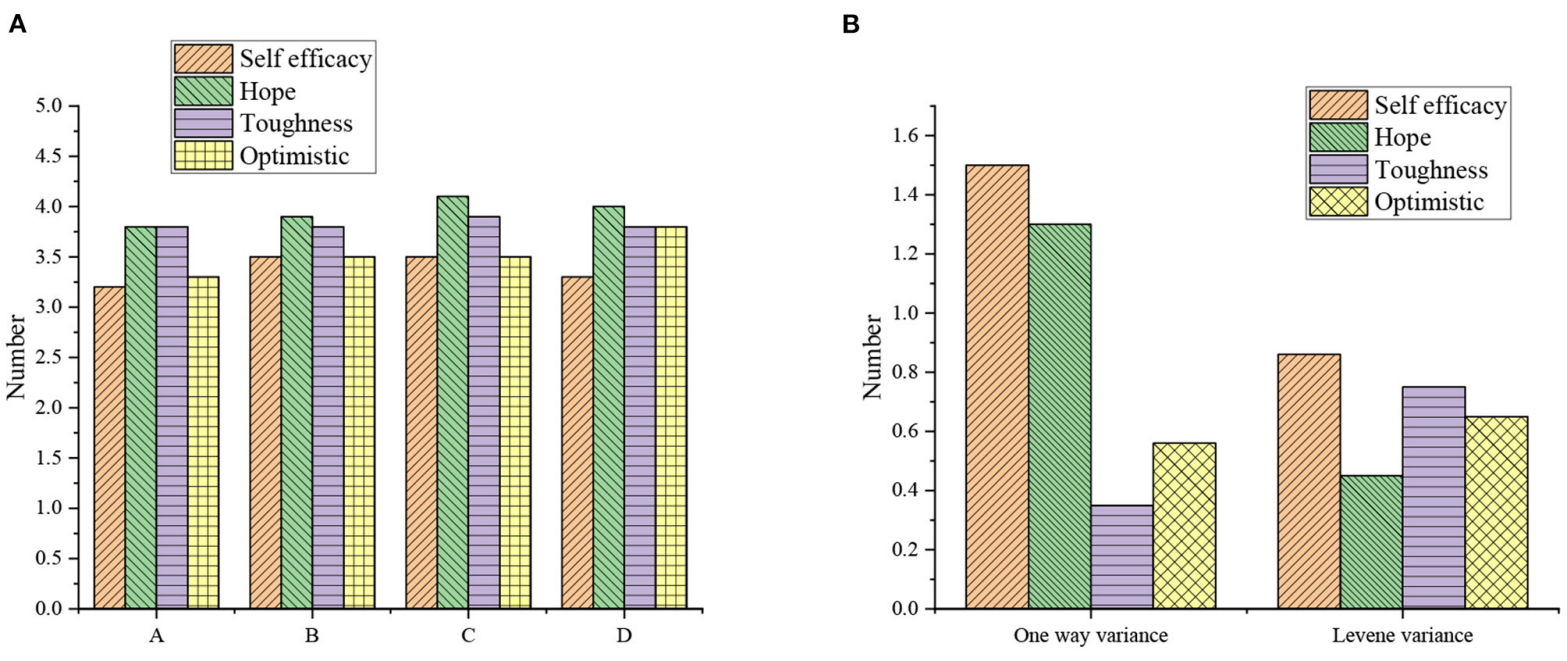
Single-factor ANOVA results of unit property on each variable [**(A)** mean; **(B)** variance test; A: state-owned enterprises; B: institutions; C: foreign enterprises; D: private enterprises; E: joint ventures].

Figure 2 shows the test results of single-factor ANOVA of unit properties on each variable.

**Figure 3:**
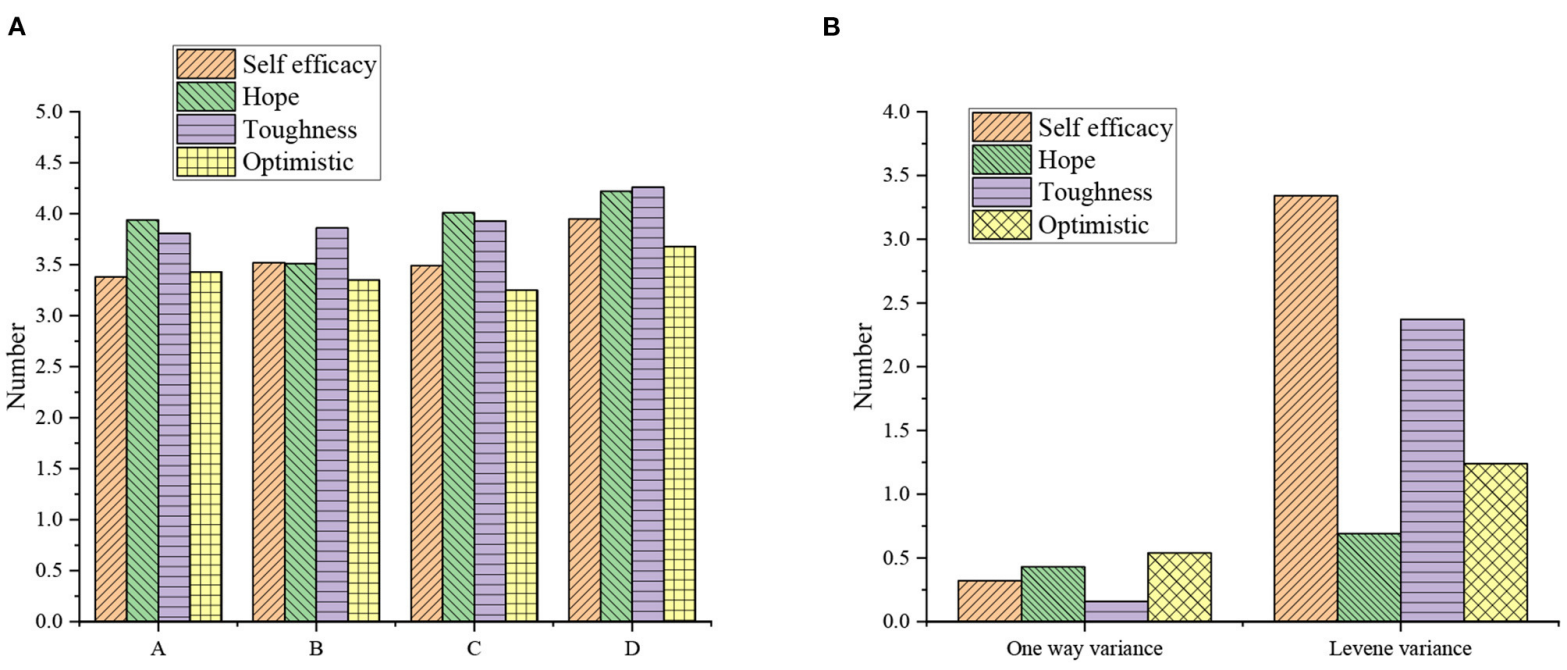
The results of single-factor ANOVA of entrepreneurial years on each variable [**(A)** mean; **(B)** variance test; A: <1 year; B: 1–3 years; C: 4–6 years; D: 7–9 years; E: over 10 years].

Figure 2 illustrates that the variances of the four dimensions (C, W, T, and O) are consistent with the homogeneity standard (*P* > 0.05), and the single-factor ANOVA shows that there is no significant difference in the unit property of C, W, T, and O (*P* > 0.05). Figure 3 displays the single-factor ANOVA results of entrepreneurial years on each variable.

**Figure 4:**
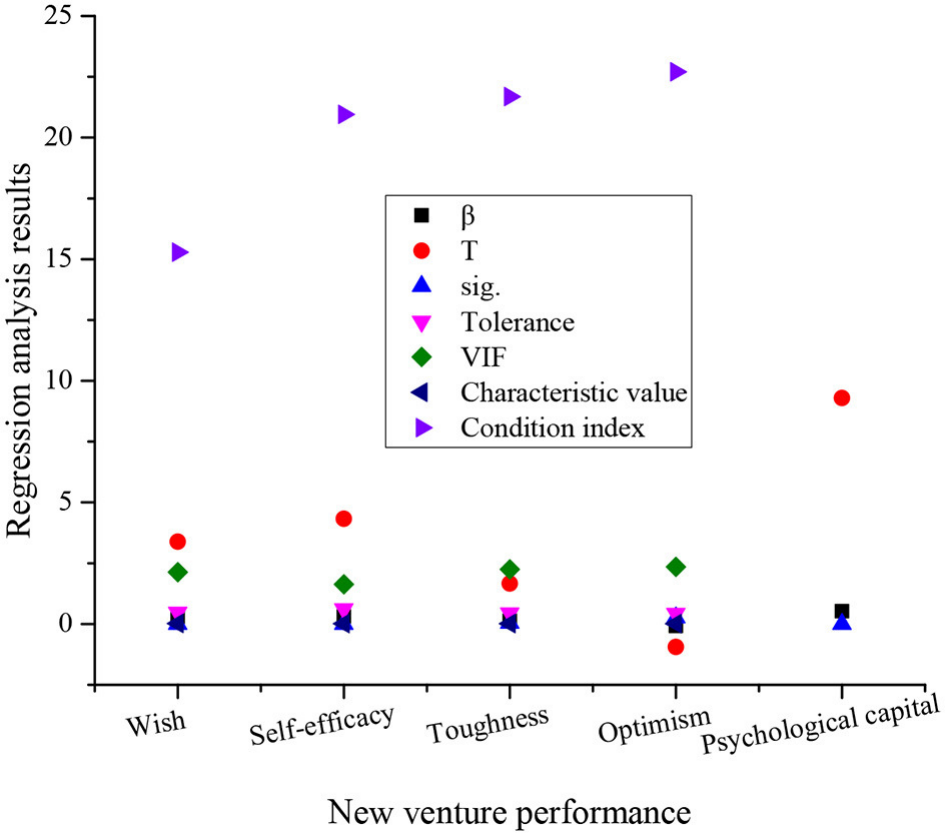
The regression analysis results of the efficacy of psychological capital of an entrepreneur on new venture performance (β denotes the Beta regression coefficient, T stands for the *T*-test result, sig. is the significant level, and VIF represents variance expansion factor).

Figure 3 demonstrates that the variances of the four dimensions (C, W, T, and O) are consistent with the homogeneity standard (*P* >0.05), and the single-factor ANOVA shows that there is no significant difference in the unit property of C, W, T, and O (*P* > 0.05). Based on the above empirical research model, the regression analysis results of the efficacy of the psychological capital of an entrepreneur on new venture performance are shown in Figure 4 and Table 5.

**Table 5:** The comparison results of subdimensions and psychological capital regression analysis.

**Outcome variables**	**R^**2**^**	**F**	**sig**.
Wish	0.326	25.509	0.000
Self-efficacy			
Toughness			
Optimism			
Psychological capital	0.294	85.403	0.000

*R^2^ represents the determination coefficient; the value of F is the F test result*.

The statistics of Figure 4 and Table 5 suggest that for new ventures in the Greater Bay Area, the psychological capital of entrepreneurs can explain 28.5% of their performance variance, the specific value of F statistics is 85.404, and the significance level at this level is very significant. Thus, the proposed H1 is verified. The influence of different dimensions on performance is different, among which, C has the largest impact. Specifically, the β value is 0.308, the value of the *T*-test is 4.328, which is very significant. The second most influential factor is W, specifically, the β value is 0.278, and the value of the *T*-test is 3.389, which is very significant. In contrast, the least influential factor on the performance of the new ventures is the T, specifically, the β value is 0.138, and the value of the *T*-test is 1.668, at a significant level. The influence of O on the performance of new ventures is not significant. Thus, the proposed hypotheses H2 to H4 are verified, while H5 is not.

**Figure 5:**
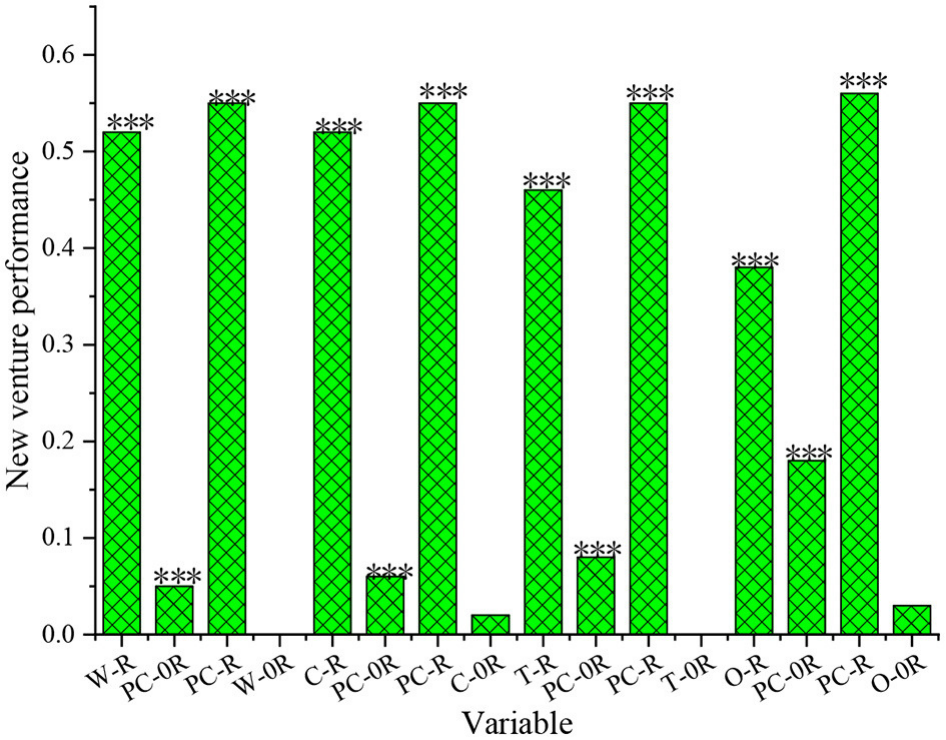
The results of utility analysis of the psychological capital of the entrepreneurs under the comparison with subdimension efficacy (R represents the multiple correlation coefficient, while 0R denotes the change corresponding to the multiple correlation coefficient, PC stands for psychological capital, and the meanings of other parameters are as defined previously), ***means *p* < 0.001, representing a significance level of 0.1%.

Furthermore, the results of the utility analysis of the comparison between the psychological capital and the four subdimensions of the entrepreneur are shown in Figure 5.

Figure 5 suggests that the overall psychological capital of the entrepreneur has a more significant effect on the performance of new ventures than any of the four subdimensions. For example, in terms of the interaction between psychological capital and C, the complex correlation coefficient between C and the performance of new ventures is 0.52, and at the same time, it is significantly below the level of 0.001. When the factor of psychological capital is introduced into the regression equation, the change value of the multiple correlation coefficient is 0.05, which is very significant. By comparison, when psychological capital is introduced into the regression equation, the change of the multiple correlation coefficient is smaller than 0.05, and the overall performance is not significant. Hence, psychological capital has a greater impact on the performance of new ventures, and so the proposed hypothesis H1 is confirmed.

**Figure 6:**
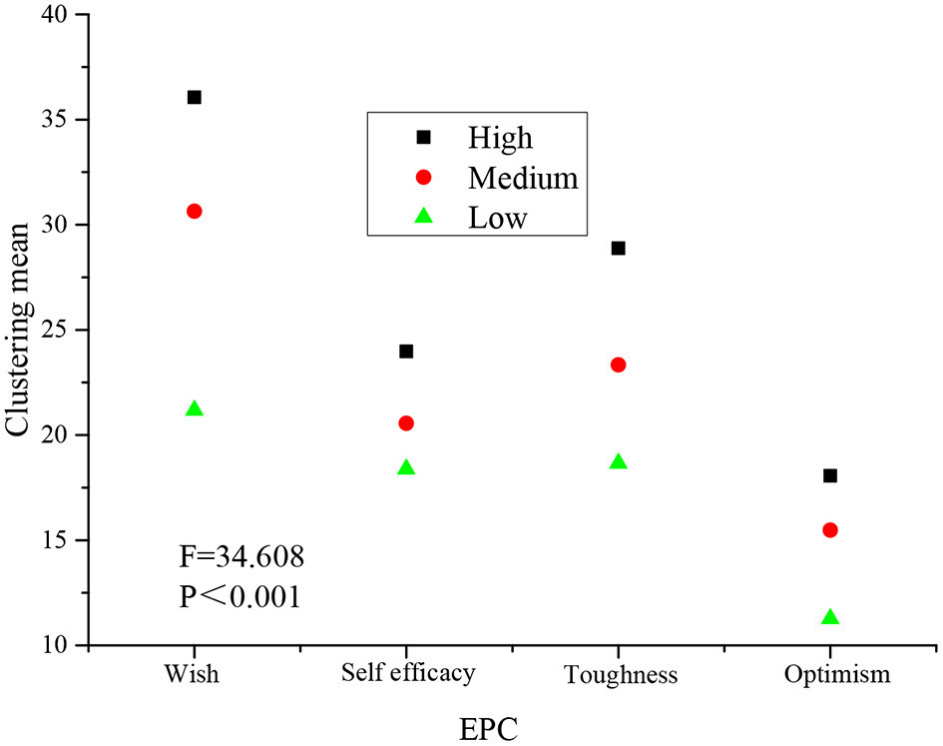
Cluster analysis results based on the efficacy of each sub-dimension of psychological capital.

Accordingly, the different efficacy of each subdimension of psychological capital is analyzed, and the cluster analysis results are shown in Figure 6.

Figure 6 reveals that among entrepreneurs in the Greater Bay Area, there are significant differences in the impact of various dimensions of the psychological capital of entrepreneurs on the performance of new ventures in the Greater Bay Area, which is manifested as the F statistical value of 34.608, and its significance is very obvious. For different levels of entrepreneurs, the overall performance of new ventures is the highest in the psychological capital group of high entrepreneurs, followed by the psychological capital group of middle entrepreneurs, and finally the psychological capital group of low entrepreneurs.

## Discussion

Here, the performance of the new venture is set as the outcome variable, and a QS is designed. The efficacy of entrepreneur psychological capital and its subdimensions are discussed. Characteristics of new venture entrepreneurs and new ventures are defined based on the statistical results of the QS, thereby laying the foundation for the analysis of the relationship between the psychological capital and new venture performance of an entrepreneur. Entrepreneurs of new ventures have a good grasp of the market situation as a whole. Most new ventures start with MSE, and the number of employees is relatively small. Compared with other factors, the dimensions of the psychological capital of an entrepreneur have a greater impact on entrepreneurship. Gong et al. (2019) found that psychological capital had a mediating role in the relationship of emotional intelligence, job performance, and job burnout, and concluded that higher psychological capital could improve employee job performance while reducing job burnout (Gong et al., 2019). Differently, however, the regional economy of the Greater Bay Area is innovatively considered here. Furthermore, the proposed five hypotheses are verified through regression analysis based on the empirical research model. Except for the subdimension of O, the three subdimensions, including C, T, and W have a positive impact on the performance of new ventures, and the overall psychological capital also has a positive impact on the performance of new ventures. On the whole, the overall impact of psychological capital on the performance of new ventures is greater than the single impact of each subdimension. Meanwhile, the impact of each subdimension on the performance of new ventures shows great differences. He et al. (2019) focused on the impact of psychological capital on the mental health and job performance of employees. The results showed that the subdimensions (including C, W, T, and O) of psychological capital have an impact on safety compliance and safety participation, thus influencing job performance. Meanwhile, the influence of each subdimension was different, and rich and flexible psychological capital intervention had a positive effect on job performance (He et al., 2019). Hence, the results obtained here are reasonable.

Greater Bay Area is an important force leading to regional innovation and economic development. The number of new ventures in this region is more than that of the other regions. Therefore, the introduction of innovative talents and related economic development measures are of great significance to reduce the brain drain and promote the development of innovative MSE. Some researchers have analyzed the importance of technological innovation in the sustainable development of industry and discussed the relationship between technological innovation and enterprise performance (Li et al., 2021). Some researchers have found that the correct use of positive psychological capital language, including W, O, T, and C, can improve the performance of crowdfunding activities (Anglin et al., 2018). Jiang et al. (2020) analyzed the urban innovation factors and found that the agglomeration of high-quality innovative talents had a positive effect on the sustainable development of the urban economy (Jiang et al., 2020). Hence, the Greater Bay Area is taken as the research background to explore the relationship between the psychological capital and the new venture performance of entrepreneurs. Here, some research results have provided support for the results on psychological capital and enterprise performance, which is different from other research works. Besides, the analysis is based on the background of regional economic development and innovation talent introduction in the Greater Bay Area, and the main analysis is of the new ventures. In short, the psychological capital of the entrepreneur has a great influence on the performance of the new ventures.

Based on the above analysis, the efficacy of the psychological capital of entrepreneurs has a positive impact on the new venture performance. Therefore, to improve venture performance, especially, new venture performance, enterprises should organize corresponding psychological capital training. Entrepreneurs should pay attention to the cultivation of their psychological quality, constantly improve their sense of self-efficacy, and cultivate toughness and optimism. In this way, entrepreneurs' interest and love for work are enhanced, and venture performance is improved driven by positive psychological capital.

## Conclusion

Here, the impact of the psychological capital of entrepreneurs on the performance of new ventures is mainly studied during regional economic development in the Greater Bay Area. Following the QS method, the introduction of psychological capital of entrepreneurs, the impact of the psychological capital of entrepreneurs on the performance of new ventures is analyzed. The statistical analysis of the QS indicates that most of the new ventures come from the financial industry, computer software industry, and education industry. The proportion of new ventures with 1–5 entrepreneurial years is the highest. Most enterprises start from MSE, and the number of employees is relatively small. Psychological capital influences entrepreneurial motivation. The results of regression analysis suggest that the psychological capital of entrepreneurs has a positive effect on the performance of new ventures. Meanwhile, three psychological capital subdimensions (C, W, and T) have a positive effect on the performance of new ventures. Overall, the impact of psychological capital as a whole on the performance of new ventures is more significant than a single subdimension. Based on the regional economic development in the Greater Bay Area, the research reveals the impact of the psychological capital of entrepreneurs on the performance of new ventures, which provides an orientation for performance improvement of the enterprises. Yet, there are also some limitations. The four dimensions of the QS are insufficient to support the research conclusion. Therefore, in the follow-up research, more multidimensional elements will be introduced to analyze and discuss.

## Data Availability Statement

The original contributions presented in the study are included in the article/supplementary material, further inquiries can be directed to the corresponding author/s.

## Ethics Statement

The studies involving human participants were reviewed and approved by Shenzhen University Ethics Committee. The patients/participants provided their written informed consent to participate in this study. Written informed consent was obtained from the individual(s) for the publication of any potentially identifiable images or data included in this article.

## Author Contributions

All authors listed have made a substantial, direct and intellectual contribution to the work, and approved it for publication.

## Conflict of Interest

The authors declare that the research was conducted in the absence of any commercial or financial relationships that could be construed as a potential conflict of interest.

## Publisher's Note

All claims expressed in this article are solely those of the authors and do not necessarily represent those of their affiliated organizations, or those of the publisher, the editors and the reviewers. Any product that may be evaluated in this article, or claim that may be made by its manufacturer, is not guaranteed or endorsed by the publisher.

## References

[B1] Alfonso U. Rodrigo F. Nidia G. Francisca L. Carlos T. Camila O.. (2018). The mediating effect of self-esteem on the relationship between perceived discrimination and psychological well-being in immigrants. PLoS ONE 13:e0198413. 10.1371/journal.pone.0198413 29927968 PMC6013095

[B2] Andersson E. Arfwidsson O. Thollander P. (2018). Benchmarking energy performance of industrial small and medium-sized enterprises using an energy efficiency index: results based on an energy audit policy program. J. Clean. Prod. 182, 883–895. 10.1016/j.jclepro.2018.02.027

[B3] Anglin A. H. Short J. C. Drover W. Stevenson R. M. Mckenny A. F. Allison T. H. (2018). The power of positivity? the influence of positive psychological capital language on crowdfunding performance. J. Bus. Ventur. 33, 470–492. 10.1016/j.jbusvent.2018.03.003

[B4] Bajwa S. S. Wang X. Duc A. N. Chanin R. M. Abrahamsson P. (2017). Start-ups must be ready to pivot. IEEE Softw. 34, 18–22. 10.1109/MS.2017.84

[B5] Cao X. Ouyang S. Yang W. Luo Y. Baochao L. I. Liu D. (2019). Transport accessibility and spatial connections of cities in the Guangdong-hong kong-Macao greater bay area. Chinese Geogr. Sci. 29, 820–833. 10.1007/s11769-019-1034-2

[B6] Diner H. Yüksel S. Martínez L. (2020). A comparative analysis of incremental and disruptive innovation policies in the European banking sector with hybrid interval type-2 fuzzy decision-making models. Int. J. Fuzzy Syst. 22, 1158–1176. 10.1007/s40815-020-00851-8

[B7] Finch J. Farrell L. J. Waters A. M. (2020). Searching for the hero in youth: does psychological capital (psyche) predict mental health symptoms and subjective wellbeing in Australian school-aged children and adolescents? Child Psychiat. Hum. D. 51, 1025–1036. 10.1007/s10578-020-01023-3 32666426 PMC7358995

[B8] Gao Q. Wu C. Wang L. Zhao X. (2020). The entrepreneur's psychological capital, creative innovation behavior, and enterprise performance. Front. Psychol. 11:1651. 10.3389/fpsyg.2020.01651 32793048 PMC7393239

[B9] Ghisellini P. Ripa M. Ulgiati S. (2018). Exploring environmental and economic costs and benefits of a circular economy approach to the construction and demolition sector. a literature review. J.Clean. Prod., 178, 618–643. 10.1016/j.jclepro.2017.11.207

[B10] Gong Z. Chen Y. Wang Y. (2019). The influence of emotional intelligence on job burnout and job performance: mediating effect of psychological capital. Front. Psychol., 10:2707. 10.3389/fpsyg.2019.02707 31920783 PMC6916327

[B11] Guo H. Cai Y. Yang Z. Zhu Z. Ouyang Y. (2021). Dynamic simulation of coastal wetlands for Guangdong-hong kong-Macao greater bay area based on multi-temporal Landsat images and flus model. Ecol. Indic., 125:107559. 10.1016/j.ecolind.2021.107559

[B12] Han X. Li Q. Wang C. Li Y. (2019). The association of occupational stress and depressive symptoms among employed persons with benign breast disease: the mediating role of psychological capital. Psychopathology 52, 1–7. 10.1159/000501164 31437833

[B13] Hasan S. Shi W. Zhu X. (2020). Impact of land use land cover changes on ecosystem service value – a case study of Guangdong, hong kong, and Macao in south china. PLoS ONE 15:e0231259. 10.1371/journal.pone.0231259 32267894 PMC7141676

[B14] He C. Jia G. Mccabe B. Chen Y. Sun J. (2019). Impact of psychological capital on construction worker safety behavior: communication competence as a mediator. J. Safety Res. 71, 231–241. 10.1016/j.jsr.2019.09.007 31862034

[B15] Jiang X. Fu W. Li G. (2020). Can the improvement of living environment stimulate urban innovation? analysis of high-quality innovative talents and foreign direct investment spillover effect mechanism. J. Clean. Prod. 255:120212. 10.1016/j.jclepro.2020.120212

[B16] Karadere M. E. Yavuz K. F. Asafov E. Y. Küükler F. K. (2019). Reliability and validity of a Turkish version of the acceptance and action diabetes questionnaire. Psychiat. Invest. 16, 418–424. 10.30773/pi.2019.02.26.2 31247700 PMC6603703

[B17] Kathrin H. Timo L. Daniel S. Julia S. Amelia M. (2018). Positive organizational behavior: longitudinal effects on subjective well-being. PLoS ONE 13:e0198588. 10.1371/journal.pone.0198588 29933367 PMC6014654

[B18] Keeeun L. Deaun G. Inchae P. Byungun Y. (2017). Exploring suitable technology for small and medium-sized enterprises (SMEs) based on a hidden Markov model using patent information and value chain analysis. Sustainability 9:1100. 10.3390/su9071100

[B19] Li F. Xu X. Li Z. Du P. Ye J. (2021). Can low-carbon technological innovation truly improve enterprise performance? the case of Chinese manufacturing companies. J. Clean. Prod. 293:125949. 10.1016/j.jclepro.2021.125949

[B20] Li T. Liang W. Yu Z. Dang X. (2020). Analysis of the influence of entrepreneur's psychological capital on employee's innovation behavior under leader-member exchange relationship. Front. Psychol. 11:1853. 10.3389/fpsyg.2020.01853 32903662 PMC7438721

[B21] Liu J. Cheng Z. Zhang H. (2017). Does industrial agglomeration promote the increase of energy efficiency in china? J. Clean. Prod. 164, 30–37. 10.1016/j.jclepro.2017.06.179

[B22] Lopez-Navarro M. A. Tortosa-Edo V. Castan-Broto V. (2018). Firm-local community relationships in polluting industrial agglomerations: how firms' commitment determines residents' perceptions. J. Clean. Prod. 186, 22–33. 10.1016/j.jclepro.2018.03.071

[B23] Lu Z. Du H. Zhao Y. Wu R. Zhang X. (2018). Correction: urban networks among Chinese cities along “the belt and road”: a case of web search activity in cyberspace. PLoS ONE 13:e0196141. 10.1371/journal.pone.0196141 29664947 PMC5903628

[B24] Michela P. (2017). Does social innovation contribute to sustainability? the case of Italian innovative start-ups. Sustainability 9:2376. 10.3390/su9122376

[B25] Rojas-Torres D. Kshetri N. (2019). Big data solutions for micro-, small-, and medium-sized enterprises in developing countries. IT Prof. 21, 67–70. 10.1109/MITP.2019.2932236

[B26] Stratman J. L. Youssef-Morgan C. M. (2019). Can positivity promote safety? psychological capital development combats cynicism and unsafe behavior. Safety Sci. 116, 13–25. 10.1016/j.ssci.2019.02.031

[B27] Sui G. Liu G. Jia L. Wang L. Yang G. (2019). Associations of workplace violence and psychological capital with depressive symptoms and burnout among doctors in Liaoning, China: a cross-sectional study. BMJ Open 9:e024186. 10.1136/bmjopen-2018-024186 31129572 PMC6538207

[B28] Taseiko O. V. Moskvichev V. V. Chernykh D. A. (2019). Regional problems in water use in Siberian industrial agglomerations. Water Resour. 46, 983–991. 10.1134/S0097807819060186

[B29] Wang D. Wang X. Xia N. (2018). How safety-related stress affects workers' safety behavior: the moderating role of psychological capital. Safety Sci. 103, 247–259. 10.1016/j.ssci.2017.11.020

[B30] Wang F. Tan Z. Yu Z. Yao S. Guo C. (2021a). Transmission and control pressure analysis of the covid-19 epidemic situation using multisource Spatio-temporal big data. PLoS ONE 16:e0249145. 10.1371/journal.pone.0249145 33780496 PMC8007114

[B31] Wang Y. Chen Y. Zhu Y. (2021b). Promoting innovative behavior in employees: the mechanism of leader psychological capital. Front. Psychol. 11:598090. 10.3389/fpsyg.2020.598090 33510678 PMC7835524

[B32] Woo C. H. Kim C. (2020). Impact of workplace incivility on compassion competence of Korean nurses: moderating effect of psychological capital. J. Nurs. Manage. 28, 682–689. 10.1111/jonm.12982 32072694

[B33] Xu W. Zhao S. (2020). The influence of entrepreneurs' psychological capital on their deviant innovation behavior. Front. Psychol. 11:1606. 10.3389/fpsyg.2020.01606 32982813 PMC7485554

